# Chatroom Sentiment Predicts Mood Improvement in Family Caregiver Mobile Peer Support Groups

**DOI:** 10.1093/geroni/igaf122.4087

**Published:** 2025-12-31

**Authors:** Noura Attili, Paulina Gutierrez-Ramirez, Maria Galvez, Liliana Ramirez Gomez, Felipe Jain

**Affiliations:** Massachusetts Institute of Technology, Cambridge, Massachusetts, United States; Massachusetts General Hospital, Boston, Massachusetts, United States; Massachusetts General Hospital, Boston, Massachusetts, United States; Massachusetts General Hospital, Boston, Massachusetts, United States; Massachusetts General Hospital / Harvard Medical School, Boston, Massachusetts, United States

## Abstract

A large proportion of family caregivers of people living with dementia experience negative impacts of caregiving on their mental health, including increased anxiety, depression, and social isolation. Online peer support groups provide an important forum for social interaction and connection for these caregivers that may help alleviate these symptoms. However, although many individuals experience peer support groups as beneficial, some find peer support groups to be unhelpful. There is little quantitative research into mechanisms that determine why some groups are effective at alleviating caregiver distress, and others are not. We aimed to study the impact of chat post sentiment within mobile application text-based chatrooms on the mental health of older adult family caregivers of dementia patients (mean age 68.5). We analyzed 3,021 chat messages using natural language processing across 29 chatrooms, which were provided as part of a randomized, controlled trial of caregiver skills with or without mentalizing imagery therapy. Outcomes included assessments of perceived stress, depressive symptoms, mindfulness, and self-compassion. Results showed that overall average positive sentiment within chatrooms, but not the sentiment of individual user posts, was significantly correlated with decreased depressive symptoms (p = 0.01), decreased perceived stress (p = 0.03), increased self-compassion (p = 0.05), and increased mindfulness (p = 0.05). These findings suggest that overall positivity within peer support chatrooms might be a mechanism by which caregivers experience clinical improvements. Increasing positivity within chatrooms might be a therapeutic target to improve caregiver mood and enhance positive psychological traits.

